# Supplementary figures and images for: Intra-articular delivery of an indoleamine 2,3-dioxygenase galectin-3 fusion protein for osteoarthritis treatment in male Lewis rats
Source: Arthritis Res Ther. 2023 Sep 18;25:173. doi: 10.1186/s13075-023-03153-0 (PMC10506271; doi:10.1186/s13075-023-03153-0)

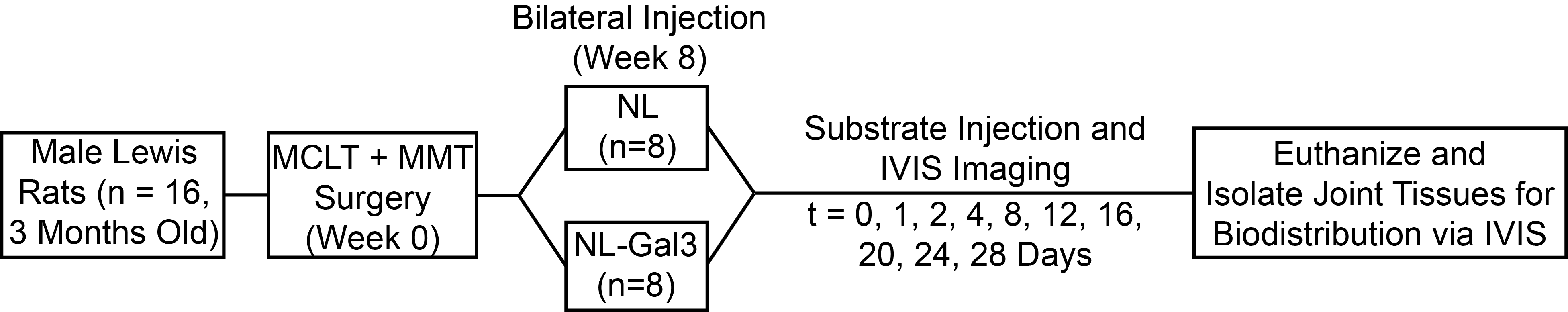

Supplement: Supplementary file 1 — Additional file 1: Supplemental Fig. 1. Flowchart describing the experimental design used to assess the joint residence and joint distribution of an enzyme after conjugation to galectin-3. Sixteen 3-month old, male Lewis Rats underwent medial collateral ligament transection plus medial meniscus transection (MCLT+MMT) surgery. At 8 weeks after MCLT+MMT surgery, rats were injected with either NanoLuc™ (NL, n=8) or NanoLuc™ galectin-3 (NL-Gal3, n=8) in both the operated and contralateral knees. Following NL or NL-Gal3 injection, rats were injected with the NL substrate, furimazine, and immediately imaged. Furimazine injections and IVIS imaging were repeated 1, 2, 4, 8, 12, 16, 20, 24, and 28 days after injection. After imaging on day 28, rats were euthanized and knees were dissected to isolate the patellar tissue, tibial tissue, femoral tissue, and meniscus for assessment of joint distribution. [file 13075_2023_3153_MOESM1_ESM.tif]

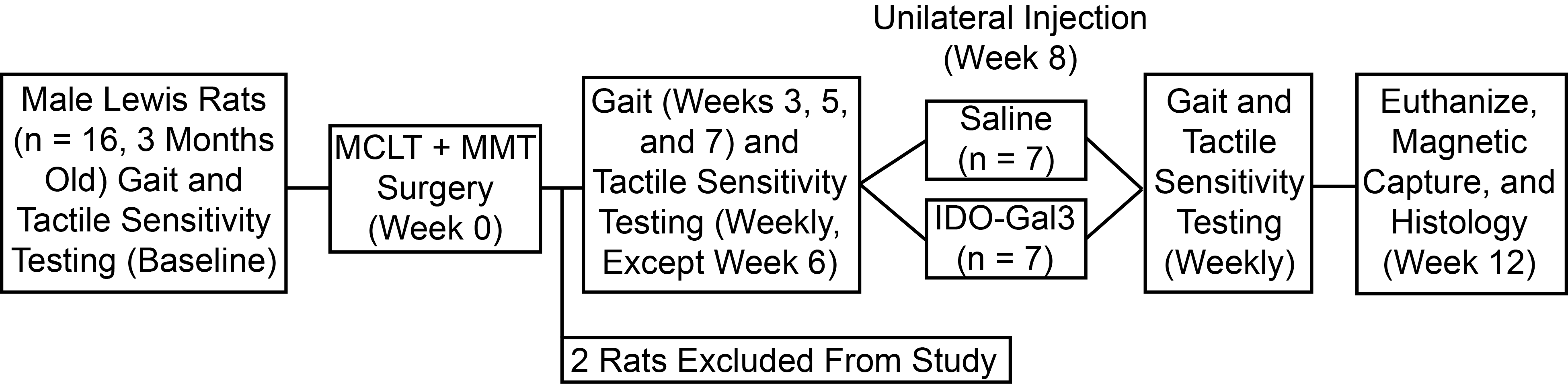

Supplement: Supplementary file 3 — Additional file 3: Supplemental Fig. 2. Flowchart describing the experimental design used to assess the ability of IDO-Gal3 to modulate OA-related pain and inflammation. Sixteen 3-month old, male Lewis rats underwent baseline gait and tactile sensitivity testing, then MCLT+MMT surgery. [file 13075_2023_3153_MOESM3_ESM.tif]

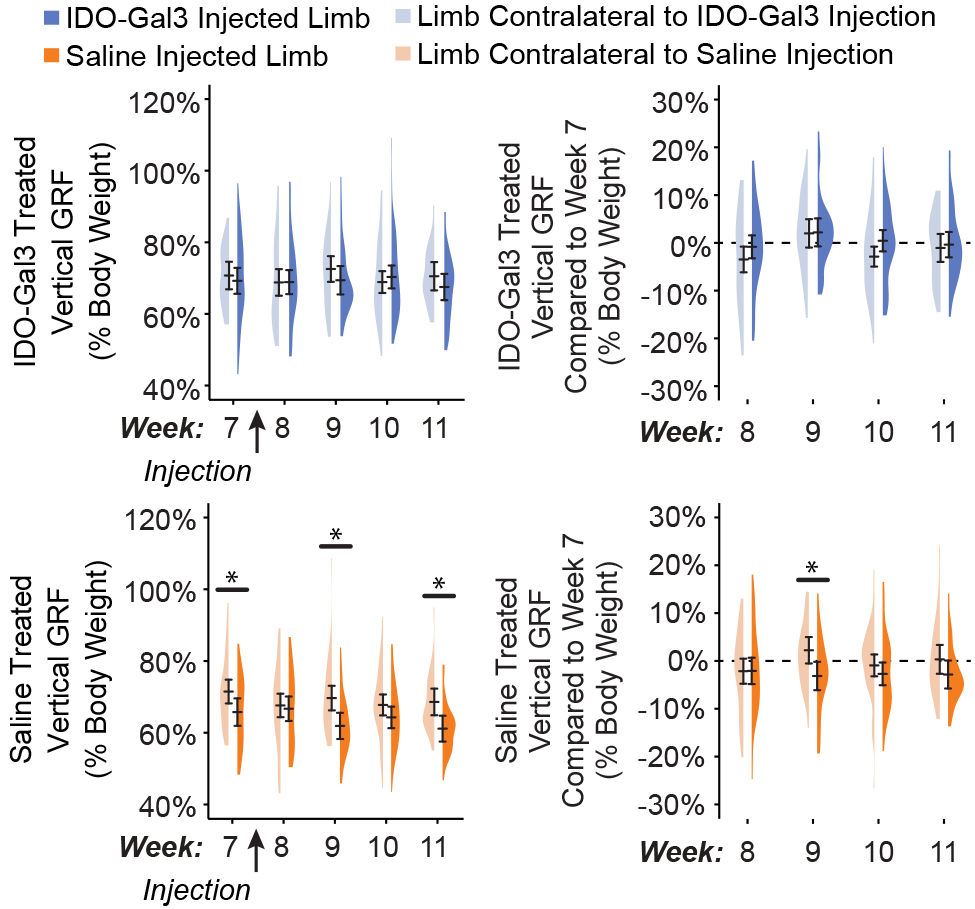

Supplement: Supplementary file 4 — Additional file 4: Supplemental Fig. 3. Peak vertical force measurements before and after intra-articular injection of IDO-Gal3 or saline into MCLT+MMT operated knees. This figure provides an alternate visualization of data presented in Fig. 4D and E, placing the left and right limbs of saline and IDO-Gal3 animals next to each other. Here, saline animals showed lower peak vertical forces in their affected limbs at week 9 and week 11 (p≤0.003). After residualizing data to the week 7 control line, differences at week 9 remained (p=0.009), while differences at week 11 were no longer significant. [file 13075_2023_3153_MOESM4_ESM.tif]

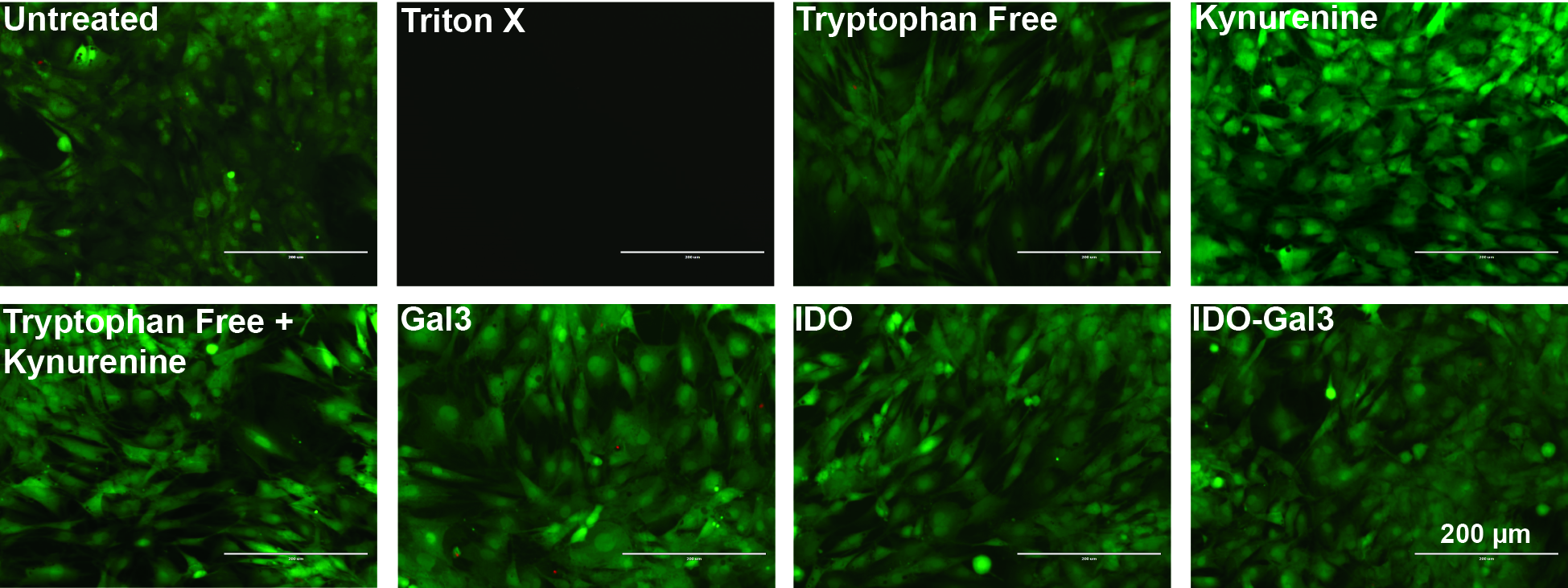

Supplement: Supplementary file 5 — Additional file 5: Supplemental Fig. 4. Synoviocyte viability after exposure to Gal3, IDO, and IDO-Gal3, as visualized with a LIVE/DEAD™ Cell Imaging Kit. In a preliminary experiment, rat synoviocyte viability after exposure to IDO-Gal3 was assessed via LIVE/DEAD™ Cell Imaging Kit (R37601, Invitrogen, Carlsbad, CA, USA) following manufacturer’s instructions. Cells were grown to 70%-80% confluence. Then, synoviocytes were incubated with Triton X-100, tryptophan free media, kynurenine-supplemented media (2080 µg/mL), tryptophan free media with a kynurenine supplement (2080 µg/mL), Gal3-supplemented media (8.3 µg/mL), IDO-supplemented media (15 µg/mL, and IDO-Gal3-supplemented media (2.37 µg/mL) (n=5/group) for 24 hrs. Concentrations of tryptophan and kynurenine were selected to be 100x greater than levels found in the OA joint (12.67 mM/mL and 8.74 µg/mL, respectively); IDO concentrations were based on necessary levels to affect 100x concentrations of tryptophan in a 24 hrs period. Synoviocyte death was not observed in tryptophan free media, kynurenine-supplemented media, tryptophan free media with a kynurenine supplement, Gal3-supplemented media, IDO-supplemented media, and IDO-Gal3-supplemented media. Treatment with Triton X-100 (positive control) elicited complete cell death. [file 13075_2023_3153_MOESM5_ESM.tif]
