# Supplementary material for: Intra-articular delivery of an indoleamine 2,3-dioxygenase galectin-3 fusion protein for osteoarthritis treatment in male Lewis rats
Source: Arthritis Res Ther. 2023 Sep 18;25:173. doi: 10.1186/s13075-023-03153-0 (PMC10506271; doi:10.1186/s13075-023-03153-0)
Supplement: Supplementary file 2 — Additional file 2: Supplemental Table 1. Estimated physical properties for NL, NL-Gal3, IDO, and IDO-Gal3. [file 13075_2023_3153_MOESM2_ESM.docx]

| Protein Name | Monomeric Mol. Weight (kDa) | Isolectric Point (pI) | Charge | Grand Average of Hydropathy | Hydrodynamic Radius (nm) |
| --- | --- | --- | --- | --- | --- |
| Nanoluciferase | 20.2 | 5.74 | -7 | 0.027 | 2.55 |
| Nanoluciferase-Gal3 | 47.4 | 6.19 | -6 | -0.247 | 3.12 |
| IDO | 47.5 | 7.3 | 0 | -0.25 | 3.12 |
| IDO-Gal3 | 72.8 | 6.99 | -1 | -0.315 | 3.59 |

**Supplemental Table 1: Estimated physical properties for NL, NL-Gal3, IDO, and IDO-Gal3**
